# 'Online' geriatric assessment procedure for older adults referred for geriatric assessment during an acute care episode for consideration of reliability of triage decisions

**DOI:** 10.1186/1471-2318-12-10

**Published:** 2012-03-20

**Authors:** Len Gray, Lucy Dakin, Steven Counsell, Helen Edwards, Richard Wootton, Melinda Martin-Khan

**Affiliations:** 1 Centre for Research in Geriatric Medicine, University of Queensland, Level 2, Building 33, Princess Alexandra Hospital, Ipswich Road, Woolloongabba, QLD 4102, Australia; 2 Indiana University School of Medicine, 1001 West 10th Street, OPW-M200, Indianapolis, Indiana 46202, USA; 3 School of Nursing and Midwifery, Queensland University of Technology, Victoria Park Road, Kelvin Grove, QLD 4059, Australia; 4 Centre for Online Health, University of Queensland, Level 3, Foundation Building, Royal Children's Hospital, Herston 4029, Australia

## Abstract

**Background:**

Comprehensive geriatric assessment has been shown to improve patient outcomes, but the geriatricians who deliver it are in short-supply. A web-based method of comprehensive geriatric assessment has been developed with the potential to improve access to specialist geriatric expertise. The current study aims to test the reliability and safety of comprehensive geriatric assessment performed "online" in making geriatric triage decisions. It will also explore the accuracy of the procedure in identifying common geriatric syndromes, and its cost relative to conventional "live" consultations.

**Methods/Design:**

The study population will consist of 270 acutely hospitalized patients referred for geriatric consultation at three sites. Paired assessments (live and online) will be conducted by independent, blinded geriatricians and the level of agreement examined. This will be compared with the level of agreement between two independent, blinded geriatricians each consulting with the patient in person (i.e. "live"). Agreement between the triage decision from live-live assessments and between the triage decision from live-online assessments will be calculated using kappa statistics. Agreement between the online and live detection of common geriatric syndromes will also be assessed using kappa statistics. Resource use data will be collected for online and live-live assessments to allow comparison between the two procedures.

**Discussion:**

If the online approach is found to be less precise than live assessment, further analysis will seek to identify patient subgroups where disagreement is more likely. This may enable a protocol to be developed that avoids unsafe clinical decisions at a distance.

**Trial registration:**

Trial registration number: ACTRN12611000936921

## Background

Comprehensive geriatric assessment (CGA) is a core procedure in specialist geriatric care. There is evidence that this process improves functional recovery, reduces morbidity and attenuates demand for long term institutional care [1]. It is central to hospital triaging processes which direct patients to formal inpatient geriatric assessment, rehabilitation, long term institutional care and complex community support programs. Geriatric consultation services are the vehicle for delivering CGA to older hospitalised patients who are not located in specialist geriatric units.

Geriatric consultation is delivered by geriatricians, gerontic nurses - alone or in partnership - sometimes with support from other allied health specialists. The availability of these aged care specialists, and the specialist services within which they work, is currently inadequate. Furthermore, the undersupply is mal-distributed, with access considerably worse in provincial cities and rural communities [2].

An innovative web based model of comprehensive geriatric assessment has been developed by The Centre for Research in Geriatric Medicine (CRGM) at the University of Queensland (UQ)[3,4]. This system utilises the interRAI Acute Care (AC) instrument, a validated assessment tool developed by the interRAI international collaborative to support evaluation of older people in acute hospital settings [5]. The interRAI AC comprises 121 individual clinical items that profile the pre-morbid and current status of the patient. The individual items have been shown to be clinically relevant and to have overall "substantial" levels of inter-rater reliability [6]. The interRAI AC incorporates screeners for common geriatric syndromes, risk assessment tools for adverse outcomes and a range of scalar measures that enable assessment of severity or dependency in the domains of pain, cognition, communication, and activities of daily living. It also includes algorithms to identify patients most likely to benefit from preventive or curative interventions, along with guidelines for further management. The instrument is administered by trained nurses who collect standardised patient data, and enter it onto a web based database. This data is then used by the geriatrician to facilitate patient assessment and provide recommendations either "online" or in conjunction with an in-person consultation. The system has also been used to support case preparation for distance assessment using video-conferencing [7].

### Research aims

This study aims to determine whether appropriate and reliable geriatric triage decisions can be made using CGA performed "online", by establishing whether agreement between "online" and "live" triage decisions is comparable (i.e. non-inferior) to inter-rater agreement for conventional "live" geriatrician assessment. It will also explore the accuracy of the procedure in identifying common geriatric syndromes, and its cost relative to conventional "live" consultations.

The key research question is: What is the level of agreement in triage decisions made by clinicians "online" and "live"?

Subsidiary research questions will provide further insight into reliability, safety and cost effectiveness:

• Is the number of patients recommended for a higher level of care (than their prior living arrangement) greater for "online" consultations?

• How often are "important" clinical diagnoses detected during live consultation that are not identified "online"?

• What is the level of agreement in detection of the common geriatric syndromes (delirium, dementia, depression) "online" compared to "live" consultation?

• What are the costs of "live" and "online" assessment procedures?

• What is the relationship between cost and effectiveness?

## Methods/Design

### Setting and participants

The study will be conducted at 3 large Australian hospitals: Princess Alexandra Hospital, Brisbane, Queensland; Box Hill Hospital, Box Hill, Victoria; and The Northern Hospital, Melbourne, Victoria. As the study is designed to evaluate the performance of the online approach when delivered by professionals with experience in using the interRAI AC instrument, at Box Hill Hospital and The Northern Hospital, where the nurses and geriatricians are new to the approach, there will be a "run in" series of 25 cases per geriatrician. This study has been approved by the Human Research Ethics Committee at each site and UQ.

All acutely hospitalised patients referred to the geriatric consultation service at each site will be considered for inclusion in the study. Patients who have previously been consented and reviewed for the study (in a former hospital episode) and those who are already known to one of the study geriatricians will be excluded.

Consent for participation in the study will be secured by the nurse administering the interRAI AC instument prior to the commencement of the consultation. Non-consenting patients will receive the "normal" geriatric consultation for the hospital site. Full demographic and limited clinical information will be secured for non-consenting subjects to enable examination of potential bias. We anticipate consent rates in excess of 95% based on previous research experience with similar subjects, and on the experience of a previous pilot study [8].

### Study design

The design aims to measure the level of agreement in triage decisions made "online" with those made for the same patient at "live" assessment, and to compare this with the level of agreement between two independent geriatricians each assessing a patient at a live consultation. The aim is to distinguish decision disagreement attributable to the online format of assessment from the 'usual' inter-geriatrician differences in clinical decision making. It is beyond the scope of this study to establish the "online-online" inter-rater reliability; this will be examined in a subsequent study and reported separately.

**Figure 1:**
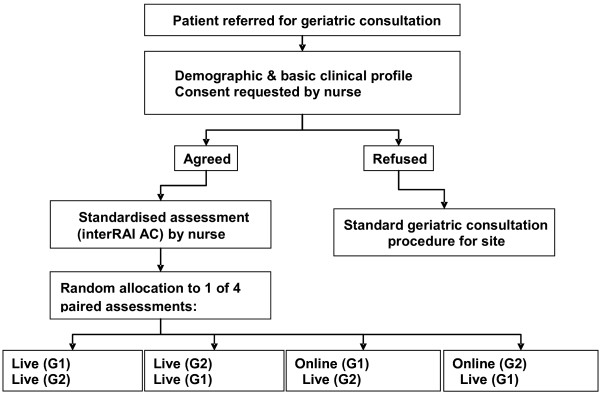
**Procedure for patient recruitment and allocation to paired consultation method**.

Each consenting patient will undergo sequential assessment by two independent geriatricians, designated "geriatrician 1" (G1) and "geriatrician 2" (G2), in one of the following randomly allocated configurations: live-live (G1-G2), live-live (G2-G1), online-live (G1-G2) or online-live (G2-G1). The patient recruitment and randomisation process is outlined in Figure 1.

The paired geriatricians will be blinded to the results of their colleague's assessment. In the case of live-live assessments the order in which the geriatricians consult the patient will be randomly allocated in order to minimise any systematic influence that the interaction of the first geriatrician with the patient may have on the second assessment.

In all cases, the interRAI AC instrument will be administered by a trained nurse. Once these findings have been entered by the nurse into the software system, a geriatrician consultation will follow. Paired geriatrician assessments will be completed in the time it usually takes for a geriatrician assessment to occur following referral to the geriatrician consultation service at that site.

For live assessments, the assessing geriatrician will have access to the interRAI AC instrument data online. The geriatrician will also be able to read the medical record. The nurse will not interact directly with the geriatrician, in order to reduce the risk of contamination of decisions when consecutive live assessments are made.

For online assessments, the geriatrician will have access to the interRAI AC instrument data online only. The geriatrician will conduct the assessment without direct contact with the nurse. The geriatrician will not have access to the medical record. However, any online test results (e.g. radiology and pathology) which are usually accessed electronically will be available to both doctors, replicating the process that would occur in a distance assessment.

### Data collection

The CGA software will collect patient level data and provide a web report for online assessors to view. At the conclusion of the assessment, the geriatricians will record geriatric triage decisions on a standard research form. Other data, such as randomisation and timing for assessments, will be collected on printed forms and entered into an appropriate database. The following data will be collected:

▪ InterRAI AC data (demographic and clinical information), collected by the nurse administering the interRAI AC instrument.

▪ Geriatrician recommendations for discharge destination: direct discharge to the community; transfer to inpatient rehabilitation or a geriatric unit; transfer to permanent residential care, including a distinction between the need for a high or low level of care (equivalent approximately to Skilled Nursing Facilities or Assisted Living in the North American context).

▪ Geriatrician prediction of discharge destination and location in 3 months: own home; residential care (high or low) or "likely to be deceased". This will provide an estimate of the ability to prognosticate using online assessment, as compared with live consultation.

▪ Presence of geriatric syndromes detected by the geriatrician (including delirium, dementia and depression).

▪ Diagnoses made by the geriatrician during assessment which were not recorded in the online report. These will be classified into those already documented in the medical record and those newly identified by the geriatrician.

▪ Costing data. Direct and indirect time contributions by relevant personnel will be recorded using time sheets. Estimates of software related costs will be calculated from data obtained from the UQ Centre for Online Health. This will be supplemented by costing information derived from existing working systems in Queensland.

▪ Three month follow up data. A research assistant will follow up each patient at three months to identify actual discharge destination and outcomes (location) at 3 months. This will be compared with the geriatrician's predictions.

### Data analysis

Agreement between the triage decisions from live-online assessments will be calculated using percentage agreement and kappa statistics, reported with 95% confidence intervals. This will also be calculated for the triage decisions from live-live assessments. Comparison of the difference in levels of agreement between the live-online group and the live-live group will be the primary measure of the reliability of the online assessment and is the basis for the sample size calculation for this project. Online assessment will be taken to be non-inferior to live assessment if percentage agreement in the live-online group is not more than 15% less than the percentage agreement in the live-live group.

The online assessment can be regarded in a similar manner to a diagnostic test, where the interest is in the number of patients recommended for a higher level of care from the online assessment compared with live assessment (i.e. false positives, specificity), and in the number of patients where important clinical diagnoses are missed in an online assessment (i.e. false negatives, sensitivity).

These measures will therefore be calculated and reported. Exploratory analyses of patient characteristics will be undertaken to examine these cases where differences of triage decision are identified. Agreement between the online and live detection of common geriatric syndromes will also be assessed using kappa statistics.

### Economic analysis

All resource use data will be collected for those who receive online assessment and those who receive the live-live assessment. Resource use will include resources associated with patient care, and resource use associated with delivery of the interventions (e.g. estimated travel time and cost of the clinician for a live assessment). The resources to be collected during the trial include staff time, diagnostic tests, inpatient stay, and patient deployment. Unit costs will be attached to each of these resource items to compare the difference in total costs between the two assessment procedures. Extrapolations, using Markov-chain MonteCarlo simulations will be undertaken to generate estimates of long-term outcomes and costs for both groups. These long-term outcomes and cost will include length of stay in hospital (using data from the observed index episode), estimates of subsequent hospitalisations, movement into residential care facilities, and mortality. The additional longer-term costs and benefits from online assessment will then be estimated to indicate any divergence between the two assessments.

### Sample size and power analysis

The outcome of interest will be the difference in percentage agreement between paired assessments for live-live arm and online-live arm for the question: "is residential care recommended?", scored as "yes" (1) or "no" (2). A non-inferiority study tests whether the percentage agreement between clinicians in an intervention arm (online-live) does not lie beyond the lower limit of an acceptable range (a one-tailed area of clinical indifference) when compared with the standard clinical practice arm (live- live).

The sample size of 288 subjects (114 online-live, 114 live-live) was identified to provide the study with power exceeding 80% to detect a non-inferiority margin of 15% for levels of agreement between online-live when compared with live-live arms for the decision "yes, residential care is recommended". This is based on an assumption of inter-geriatrician agreement of 60% for geriatric triage decisions [8], allowing a two-sided type 1 error rate of 5%. With a 15% allowance for attrition, the adjusted sample size is 268 (i.e. 228/(1-0.15)). After rounding, 270 cases will be required in total between all sites.

### Randomisation

The following process for randomisation will be carried out by the statistician. The < ralloc > command will be used to generate 2 separate randomisations in Stata version 10.1. These allocations will then combined to create the individual patient allocation to geriatrician/intervention and stored in individually numbered opaque envelopes for each site. Initially, patients will be equally randomised to the 4 intervention arms in blocks of 4 and 8. Patients will then be equally randomised to the 2 intervention arms (geriatricians 1 and 2) in blocks of 2 and 4. Due to the constraint that a maximum of two patients are to be randomised on any one day and the same geriatrician must not be used for both, the second of the two patients is deterministically allocated to the doctor who does not attend to the first of the two patients. Allocations will be approximately balanced across 3 studies centres.

## Discussion

This study is designed to identify the strengths and weaknesses of an online method of CGA. It seeks to identify whether consistent, reliable and safe recommendations can be made online. Even if the online decisions prove to be deficient to some extent, the study will allow effectiveness to be weighed against cost and may develop a methodology to identify patients who can be safely assessed online as opposed to those who require in-person consultation. A clinical algorithm would then enable online clinical reviewers to quickly determine whether a case requires live assessment. If successful, online CGA has the potential to increase access to geriatrician expertise, especially in rural and remote locations or areas of urban isolation such as residential care.

## Competing interests

The authors declare that they have no competing interests.

## Authors' contributions

LG led the design of the protocol and contributed to the preparation of the draft. LD contributed to the preparation of the draft. SC, HE and RW participated in the design of the protocol and contributed to the preparation of the draft. MMK assisted LG in conceptualisation and design of the study, participated in the design of the protocol and contributed to the preparation of the draft. All authors read and approved the final manuscript.

## Pre-publication history

The pre-publication history for this paper can be accessed here:

http://www.biomedcentral.com/1471-2318/12/10/prepub
